# The Phenomenon of Social and Pastoral Service in Eastern Slovakia and Northwestern Czech Republic during the COVID-19 Pandemic: Comparison of Two Selected Units of Former Czechoslovakia in the Context of the Perspective of Positive Solutions

**DOI:** 10.3390/ijerph19042480

**Published:** 2022-02-21

**Authors:** Patrik Maturkanič, Ivana Tomanová Čergeťová, Roman Králik, Ľubomír Hlad, Marie Roubalová, Jose Garcia Martin, Viliam Judák, Amantius Akimjak, Lucia Petrikovičová

**Affiliations:** 1 Faculty of Roman Catholic Theology of Cyril and Methodius, Comenius University Bratislava, 81458 Bratislava, Slovakia; patrikmat@seznam.cz (P.M.); judak1@uniba.sk (V.J.); 2 College of Applied Psychology, 41155 Terezín, Czech Republic; cergetova.ivana@gmail.com; 3 Department of Russian Language, Peoples’ Friendship University of Russia (RUDN), 117198 Moscow, Russia; roman.kralik73@gmail.com; 4 Department of Social Works, Faculty of Theology, Catholic University in Ružomberok, 03401 Ruzomberok, Slovakia; amantius.akimjak@ku.sk; 5 Department of Religious Studies, Faculty of Arts, Constantine the Philosopher University in Nitra, 94901 Nitra, Slovakia; lhlad@ukf.sk; 6 Department of Biblical and Jewish Studies, Hussite Theological Faculty, Charles University in Prague, 14000 Prague, Czech Republic; marie.roubalova@htf.cuni.cz; 7 Department of Sociology, Faculty of Political Sciences and Sociology, University of Granada, 18001 Granada, Spain; jgarciamartin@ugr.es; 8 Department of Geography, Geoinformatics and Regional Development, Faculty of Natural Sciences and Informatics, Constantine the Philosopher University in Nitra, 94901 Nitra, Slovakia

**Keywords:** social and pastoral service, religious affiliation, pandemic situation

## Abstract

This study seeks to explain the differences in the perception of social and pastoral service after the first and second wave pandemic in 2020 among the inhabitants of two neighbouring states, both parts of the former unified Czechoslovakia. Our research study compares subjective perception, needs, and participation among inhabitants of eastern Slovakia and north-western Czech Republic in social and pastoral service during the COVID-19 pandemic. The research sample consisted of a healthy population from the Czech Republic (*n* = 496) and Slovakia (*n* = 484) over 16 years of age, of which 63% (*n* = 617) were women and 37% (*n* = 363) men. The level of education ranged from primary to postgraduate. The research sample consisted of 623 (63.6%) participants with religious affiliation and 357 (36.4%) without religion. The level of perception, needs, and participation of the participants in social and pastoral service was obtained based on a non-standardised questionnaire. The results of our study confirmed several differences in the areas studied.

## 1. Introduction

At the end of 2019, the outbreak of COVID-19 occurred, causing severe risks within the EU associated with discrimination, poverty, and the social exclusion of many sections of the population [1,2,3,4,5]. The pandemic paralysed the world and the European economy. Day by day, many sections of the population have been at risk of poverty and social exclusion [6,7]. The countries of former Czechoslovakia did not escape this situation either [8]. Therefore, we can agree with Kondrla et al. that “the need for systematic social work and community social work is proving to be relevant; all the more so is the need for social workers who would be willing to bring positive elements into this specific environment” [9]. Relevant topics in the context of this goal appear to be, for example, social issues about the education and self-education of social workers and social work assistants, who have found themselves in the middle of a pandemic. The appropriate ways to “help them strengthen the key competences and basic skills, which stand (and fall) on an individual’s voluntary commitment, based on a high level of motivation and proactivity” [10]. Thus, we might agree with Ref. [11], who pointed out the necessity of “the formation of educational values (i.e., pragmatic-instrumental perception of a person’s education), work values (a mixture of optimism and pessimism in assessing the possibility of finding employment), characteristics of interpersonal interaction (high credit for trust in other people), general value priorities (for example attitudes towards education) and so on”. In the context of these ideas, one of the critical topics that we cannot ignore from a global perspective seems to be media’s influence on social cohesion between people, which some experts have proven through their research [12]. In parallel with these findings, we examine the consequences of social exclusion, discrimination, or stereotyping, which happen due to the media [13].

Similarly significant issues seem to concern the “correct balance between human needs and the carrying capacity of all human needs”, including the carrying capacity of all human ideas and requirements, of course, in a regional and global context [14,15]. One of the critical topics that we cannot ignore from a global perspective is social issues, which undoubtedly affect every human society [16]. After all, the prioritisation of interest in the weak and vulnerable should show in a balanced, i.e., mature way, so that we are not indifferent to any level of human suffering and pain, both physical and mental.

Let us look at this problem from a different perspective, flip the coin, and ask about the positive significance of this constant truth. First, suffering reminds a person of the vulnerability of their existence, and therefore, in this extraordinary way, they encourage themselves to plan their life responsibly. The philosophy of various systems shows that this widespread phenomenon puts man on the plane of a wise, and therefore humble, search for himself. Let us recall the biblical story that describes Job’s suffering. We can see this righteous man enduring a series of degrading trials, even the abandonment of God himself, while remaining faithful to him. His reward is a long and happy life, as lovingly described in the Old Testament text. The second positive aspect of human suffering can be discovered in its further anthropological understanding: man’s attitude towards his neighbour. These are often our sick parents, life partners, and even our children [17]. Here is a moment when we see how helpless we are, even though we have often considered ourselves to be a force in the world.

On the contrary, this human helplessness should lead us to a compassionate attitude in befriending others and forgiving things in these most difficult moments that we might otherwise persist with. We should agree with the statement that human pain sometimes has literally “served”, that is, it has become the meaning and value of higher goals that transcend the physical world in the lustre of eschatological immortality, as the Christian worldview teaches us [18].

In this spirit, we can speak of social and pastoral care, which in the needy person exceeds professional social and practical help, which is either closely or distantly related to the faith of the needy, to that of the helper [19] or to that of both parties. So, what does this service mean in connection with the social field? We do not want to look for any additional element of ordinary parish pastoral care, but above all for personal, humane accompaniment, wherein with a spirit of respect for mutual freedom we help the needy manage their life situation in the best and most dignified way. The word “accompaniment” seems very important. Accompaniment eliminates both manipulation and indifference. For if we take a person seriously in their completeness, then we cannot separate either their physical or mental need or their dimension of faith from their life. If we respect the person in their uniqueness, we respect their path of faith and accompany them on it. We refer here to the individual care of a person who finds themselves in a non-standard life situation where they cannot manage it alone and need help. From this encounter, which respects the authenticity of everyone who enters it and its originality, the authenticity of the Christian service is born.

We must also note that after the coup in 1948, the communist regime systematically persecuted the church and destroyed its structures. The cruelty of the system was felt especially by the order. The communist regime tried to build people’s mistrust and hatred towards everything religious. There has been no religious freedom as we had known it since 1989, when Czechoslovakia became a free country again [20,21].

Thus, we can come to the idea of pastoral service in social work, which can serve a person who explicitly believes and those who either do not communicate about matters of faith or reject this topic, without being indifferent in terms of faith or vice versa. In this research, social and pastoral service is characterised as an emotional and spiritual support model that can be found in many cultures and traditions. Our modern context has described it as an individual and patient model in which trained social and pastoral carers support people in their pain, loss, and anxiety, and their triumphs, joys, and victories. Social work was an excuse to push faith and acceptance as a condition for willingness to take care of the client [10]. Social and pastoral service is thus a central research topic, and initial information about the functioning of society is beginning to emerge [22,23].

## 2. Materials and Methods

Our research study compares the subjective perception, needs, and participation of inhabitants in eastern Slovakia and north-western Czech Republic in social and pastoral service during the COVID-19 pandemic. In 2021, the level of long-term unemployment in Slovakia was 6.8%. A total of 56% of the population of Slovakia professed the Roman Catholic faith in 2021. The level of long-term unemployment in the Czech Republic in 2021 was 3.5%. During the census in Bohemia in 2021, in response to religious belief, 18.7% of those who filled out the questionnaire declared that they were believers and belonged to a church or religious society. The respondents without religious faith accounted for more than two thirds (68.3%) of the respondents. Answering the question on religious belief was not obligatory; 30.1% of people left it blank, while in the 2011 census, 44.7% left it blank. The level of long-term unemployment in the Czech Republic in 2021 was 3.5%. During the census in Bohemia in 2021, in response to religious belief, 18.7% of those who filled out the questionnaire declared that they were believers and belonged to a church or religious society. The respondents without religious faith accounted for more than two thirds (68.3%) of the respondents. Answering the question on religious belief was not obligatory; 30.1% of people left it blank, while in the 2011 census, 44.7% left it blank.

We obtained data from research participants through an online distributed non-standardised structured questionnaire with several open and closed questions. In addition, several socio-demographic issues were included in the inquiry. The primary research question of this study was: Are there differences in the level of subjective perceptions, needs, and participation in social and pastoral service between the inhabitants of eastern Slovakia and north-western Czech Republic? The secondary question in our research was: Are there differences in the level of subjective perception, needs, and participation in social and pastoral service in the research sample based on gender, age, level of education, the size of the municipality in which they live, and religious affiliation? The study aimed to obtain data on the differences in the research sample that were expected in each area.

The research sample consisted of a healthy Czech and Slovak population, with 50.6% from Czech Republic (*n* = 496) and 49.4% from Slovakia (*n* = 484). The distribution of the research sample by gender was 63% women (*n* = 617) and 37% men (*n* = 363). The research participants were divided into five age groups. In the group up to 18 years were 12.2% of participants (*n* = 120), in the group from 18 to 25 years were 11.5% (*n* = 113), from 26 to 40 were 19.2% (*n* = 188), from 41 to 59 were 39.8% (*n* = 390), and over 60 years were 17.2% (*n* = 169) of participants. With regard to the level of education attained, the research sample was focused mainly on university graduates. The study identified 127 people with basic education (13%), 368 people with secondary and higher vocational education (37.5%), and 485 with university and postgraduate education (49.5%). We divided the research participants based on their religious affiliation. In total, 63.6% of participants determined they had an affiliation to religion (*n* = 623), and 36.4% of participants were without religious affiliation (*n* = 357). The participants were also divided based on the size of the municipality from which they came. A total of 28.4% of the research participants came from a village (*n* = 278), 16.5% came from a small town of up to 10,000 inhabitants (*n* = 162), 32.2% came from a town of 10,000 to 50,000 (*n* = 316), 14.0% participants came from a town of 50,000 to 100,000 (*n* = 137), and 8.9% came from a town of 100,000 inhabitants (*n* = 87).

The research was carried out in the second quarter of 2021 after the second wave of the COVID-19 pandemic. The research participants were contacted directly via personalised mail through the online form. They had the opportunity to forward the questionnaire to others interested in participating in the research who met the research criteria. The data were evaluated in the following period with anonymity. Research participants were asked to answer questions and receive instructions. By completing the questionnaire and answering all the questions, the participants confirmed their consent to participate in the survey. No time limit was set during administration.

The collection of the data was carried out anonymously. The data were evaluated using a statistical program SPSS (Version 23 for Windows, IBM, Armonk, NY, USA). Data were evaluated using descriptive and inferential statistics, using mean, standard deviation, and minimum and maximum scores. The results were examined through descriptive statistics, contingency tables, and a contingency table variability test. The variable independence test assumes that the random variables X and Y are independent, so the values of one variable do not affect the values of the other variable. The dependence between variables can be either one-sided (asymmetric) or mutual (symmetric), where both variables interact. Pearson’s chi-square test was used to test the independence of two categorical variables in the PivotTable, regardless of the direction of their dependence. The null hypothesis of this test assumes that both variables are independent of each other. We tested this null hypothesis at the determined level of significance α, that the variables are independent, as opposed to the alternative that there is a dependence between the variables. We wrote the hypotheses as follows:

(1) $$\begin{array}{c} H_{0}:\;n_{ij}=\frac{n_{i}*n_{j}}{n}\\ H_{1}:\;n_{ij}\neq \frac{n_{i}*n_{j}}{n} \end{array}$$

where *n_ij_* denotes the frequencies in the PivotTable, *i* = 1, 2, …, *r* denotes the categories of the variable X and *j* = 1, 2, …, and *s* denotes the categories of the variable Y.

The test criterion χ^2^ is defined as:

(2) $$\chi^{2}=\overset{r}{\underset{i=1}{\sum }}\overset{s}{\underset{j=1}{\sum }}\frac{{\left(n_{ij}-n_{ij}^{\prime }\right)}^{2}}{n_{ij}^{\prime }}$$

where χ^2^ ≈ χ^2^ [(*r* − 1) (*s* − 1)]. The larger the differences between the categories of the examined variables, the larger the test criterion χ^2^.

The prerequisite for using this test is that theoretical frequencies of less than five observations make up less than 20% of the pivot table fields. Individual categories of variables can be combined to meet this assumption.

## 3. Results

We were the first to evaluate data obtained from a healthy Slovak and Czech population during the 2021 pandemic based on descriptive statistics. In the following section, we present frequency tables in bar graphs that describe the research results. In order to answer our first research question, we divided the research participants according to their place of residence into the first group of participants from eastern Slovakia and the second group of participants from north-western Czech Republic.

**Figure 1:**
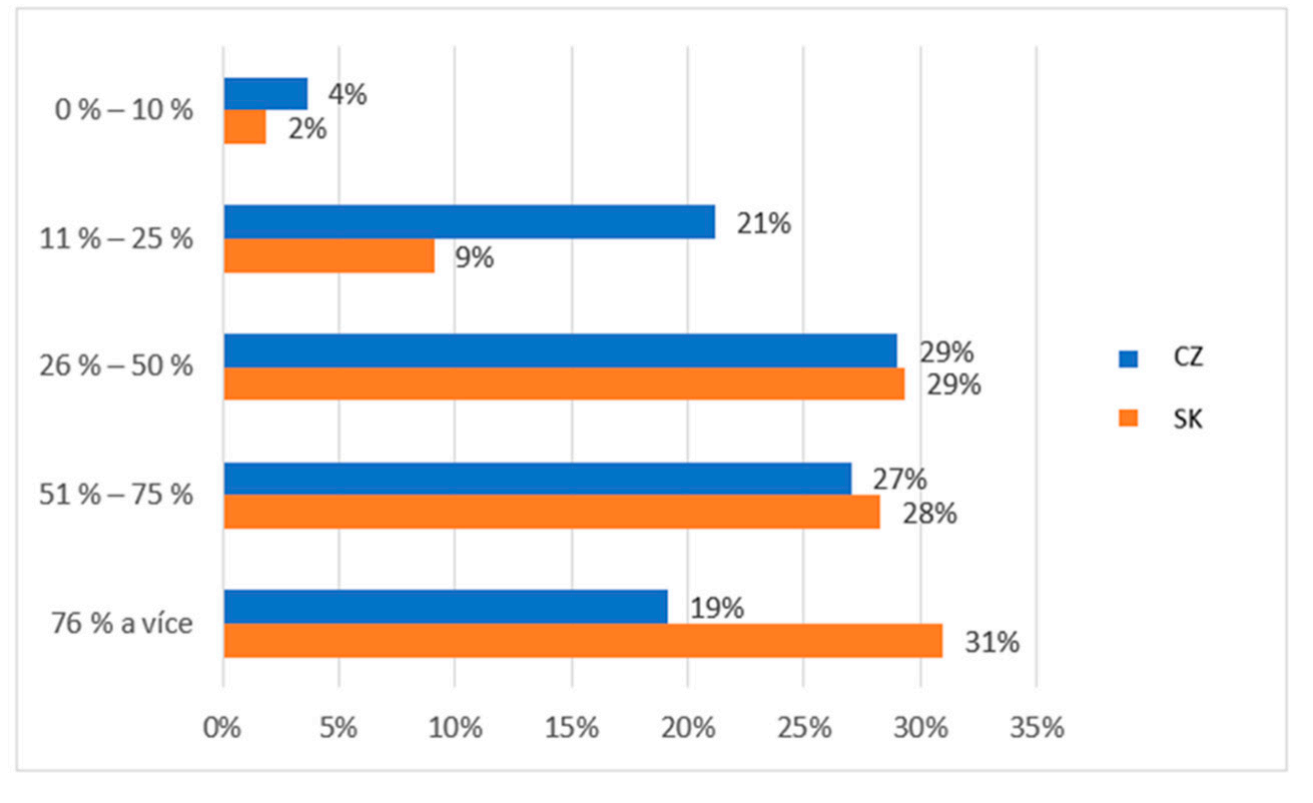
Descriptive statistics of the participants’ subjective needs of social and pastoral service (comparison of the healthy population of eastern Slovakia and north-western Czech Republic). Legend: CZ—healthy population of north-western Czech Republic, SK—healthy population of eastern Slovakia. Source: own resource.

The first question we asked the participants regarding their need for social and pastoral service was as follows: How would you express the percentage of the need for social and pastoral service in the society in which you live? Participants had the opportunity to choose from five options differing in percentage (0–10, 11–25, 26–50, 51–75, and 76% and more). The research results in Figure 1 show a different perception of social and pastoral service needs between the groups compared. As many as 31% of Slovaks subjectively perceived the need for social and pastoral service during a pandemic at a level above 75%. Compared to the Czechs, there was a significant difference of opinion of 12% in this case. This difference is shown in the Czechs in the percentage between 11 and 25%; up to 21% of members of this group thought that the percentage needed was at this lower level.

**Figure 2:**
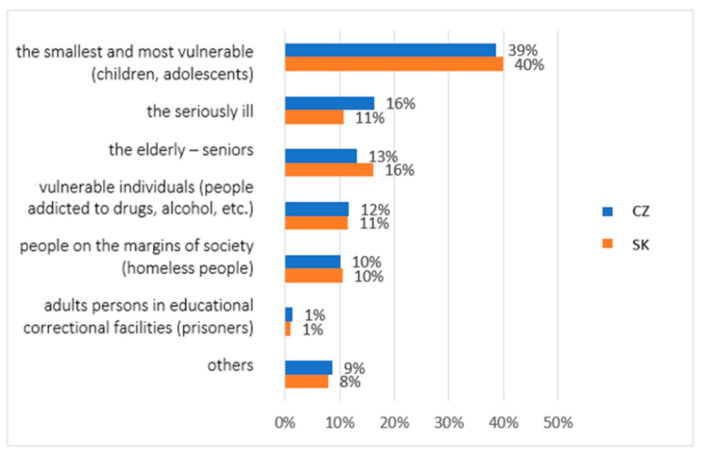
Descriptive statistics of the participants’ subjective perception of social and pastoral service (comparison of the healthy population of eastern Slovakia and north-western Czech Republic). Legend: CZ—healthy population of north-western Czech Republic, SK—healthy population of eastern Slovakia. Source: own resource.

In the second question, we asked the research participants for their opinion regarding the target group of social and pastoral service: In your opinion, which group of people deserves the most “social attention”? Research participants were able to identify several options: the youngest and most vulnerable (children, adolescents), the seriously ill, the elderly, socially vulnerable individuals (people addicted to drugs, alcohol, etc.), people on the margins of society (homeless people), adults in educational correctional facilities (prisoners), and other groups. From the results shown in Figure 2, we can see no significant differences in opinion between the groups. The most endangered group is the youngest and most vulnerable—young children and adolescents (40% of Slovaks and 39% of Czechs identified as deserving “social attention”). The groups of the seriously ill (11% of Slovaks and 16% of Czechs) and seniors (16% of Slovaks and 13% of Czechs) were identified as the second and third groups requiring “social attention”. These two “social attention” groups are the only groups in which we recorded subtle differences of opinion between the surveyed research groups. For the other groups—vulnerable individuals, people on the margins of society, prisoners, and others—the answers were very similar in both research groups. The group of vulnerable individuals was marked by 11% of Slovaks and 12% of Czechs. People on the margins of society were marked by 10% of both groups. A total of 8% of Slovaks and 9% of Czechs chose the group “others”, and only a total of 2% of participants considered prisoners to be persons in need of social and pastoral service.

**Figure 3:**
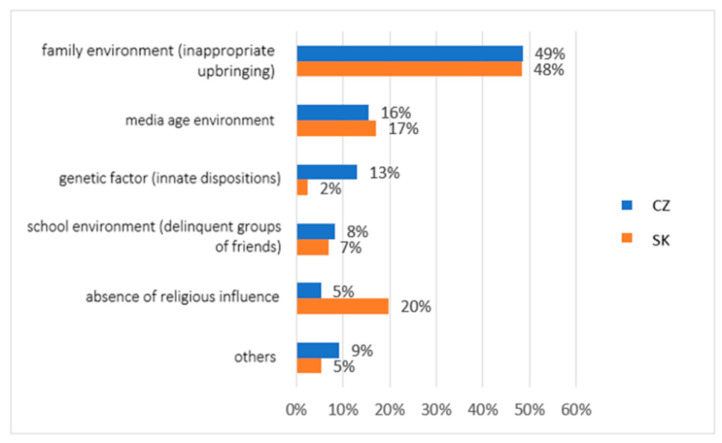
Descriptive statistics of the participants’ subjective perception of the reason for people’s deviant behaviour (comparison of the healthy population of eastern Slovakia and north-western Czech Republic). Legend: CZ—healthy population of north-western Czech Republic, SK—healthy population of eastern Slovakia. Source: own resource.

Another question in our research concerned the subjective perception of the reason for people’s deviant behaviour. The question was as follows: What do you see as the primary cause in some people’s social-deviating behaviour? Respondents had the opportunity to choose from the following options: family environment (inappropriate upbringing), media age environment, genetic factor (innate dispositions), school environment (delinquent groups of friends), absence of religious influence, and others. From the results shown in Figure 3, we can see that almost half of all participants (a total of 48% Slovaks and 49% Czechs) think that the cause of deviant behaviour is an unsuitable family environment. Both groups examined were similar in their responses related to several factors—media age environment (this answer was marked by 17% Slovaks and 16% Czechs), school environment (answered by 7% Slovaks and 8% Czechs), and others (5% Slovaks and 9% Czechs chose this option). However, we perceive differences in opinions regarding two possibilities, namely the genetic factor, where only 2% of people from Slovakia marked this answer and up to 13% from Czech Republic marked it. On the contrary, in the case of factors related to the absence of religious influence, up to 20% of Slovaks and only 5% of Czechs indicated this possibility.

**Figure 4:**
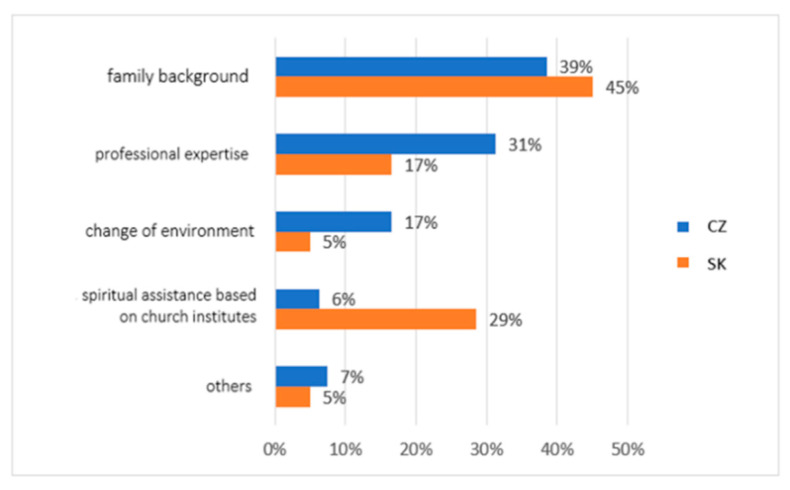
Descriptive statistics of the participants’ subjective perception of effective support for socially vulnerable people (comparison of the healthy population of eastern Slovakia and north-western Czech Republic). Legend: CZ—healthy population of north-western Czech Republic, SK—healthy population of eastern Slovakia. Source: own resource.

The fourth question in our research concerned the subjective perception of the most effective support for vulnerable groups. We asked: In your opinion, which of these forms of help is the most effective support for these socially vulnerable people? Research participants had the opportunity to choose the following forms of assistance: family background, professional expertise, change of environment (residence, employment, friends, etc.), spiritual assistance based on church institutes, and others. In the answers to this question, we can see significant differences of opinion in the compared groups (Figure 4). The most prominent answer was a form of help from the family background. In total, 45% of Slovaks and 39% of Czechs chose this option. Furthermore, research participants from Slovakia identified spiritual assistance based on church institutes as the second and third most crucial form of support (29%), followed by professional expertise (17%). The last were two forms of assistance at the level of 5%, namely change of environment and others. In the case of Czech research participants, they identified professional expertise as the second most important form of assistance (31%). The third most important form was change of environment (17%), then other ways (7%), and finally, spiritual assistance (only 6%).

**Figure 5:**
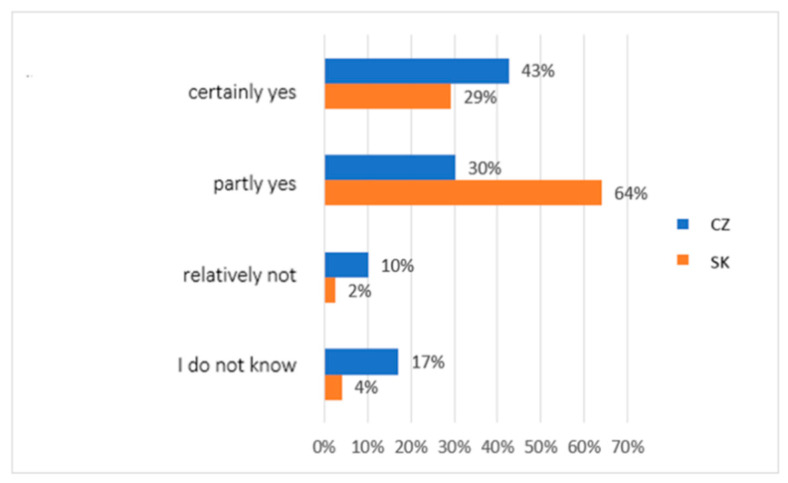
Descriptive statistics of the participants’ subjective perception of the role of religious organisations in the social and pastoral service (comparison of the healthy population of eastern Slovakia and north-western Czech Republic). Legend: CZ—healthy population of north-western Czech Republic, SK—healthy population of eastern Slovakia. Source: own resource.

The next question in our research concerned the subjective perception of the role of religious organisations in the social and pastoral service. We asked: Do religious organisations’ social and pastoral service play a social role in your opinion? Research participants were able to choose the answers on the scale of importance of the social role from the following: certainly yes (this area belongs to their priority mission), partly yes (these activities are also in charge of other organisations), relatively not (I see their mission in other dimensions of social life), or I do not know. The results in Figure 5 again show differences of opinion between the groups. In total, 64% of the surveyed Slovaks perceived this role of church organisations in part, and 29% clearly as a social role. Among Czechs, the opinion was significantly different, as 43% perceived the social role of church organisations in social and pastoral service, while 30% perceived it in part. Together, only 6% of Slovaks surveyed did not have church organisations associated with this social role or could answer. In the case of Czechs, this cumulatively made up 27% of respondents.

**Figure 6:**
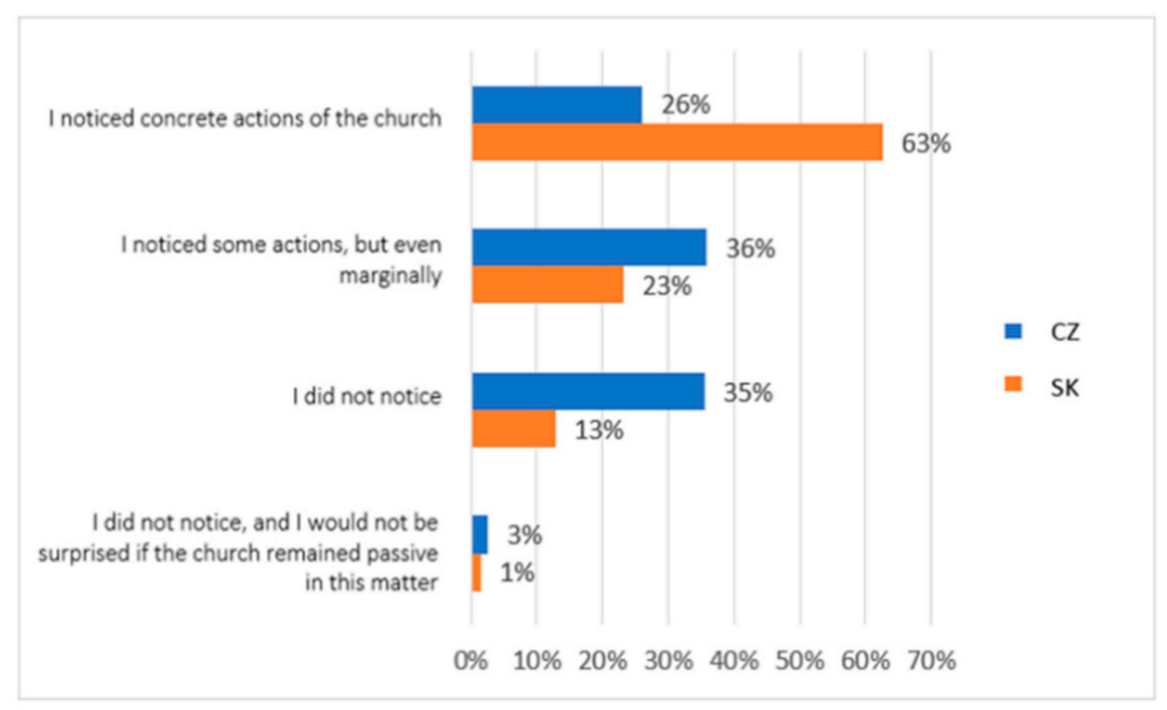
Descriptive statistics of the participants’ subjective perception of activities in social and pastoral service (comparison of the healthy population of eastern Slovakia and north-western Czech Republic). Legend: CZ—healthy population of north-western Czech Republic, SK—healthy population of eastern Slovakia. Source: own resource.

In the following question, we asked research participants about the subjective perception of social and pastoral service in their surroundings: Did you notice people in your neighbourhood who belong to a church institution that actively participated in the social assistance of others? The answers to this question concerned the perception of activities in several ways: (1) I noticed concrete actions of the church, (2) I noticed some actions, but even marginally, (3) I did not notice, (4) I did not notice, and I would not be surprised if the church remained passive in this matter. The research results shown in Figure 6 show a different subjective perception of the church’s activities in social and pastoral service in the target groups studied. In the case of a group of participants from Slovakia, they significantly perceived specific activities (63%) or marginally perceived them (23%). A total of 13% did not notice any activity. Only 1% also rated the church negatively. A total of 26% of the Czech participants in the research noticed the activity of the people in the church, and 36% partly noticed it. As many as 35% of the participants did not notice any activity, and 3% also negatively assessed the inactivity of the church.

**Figure 7:**
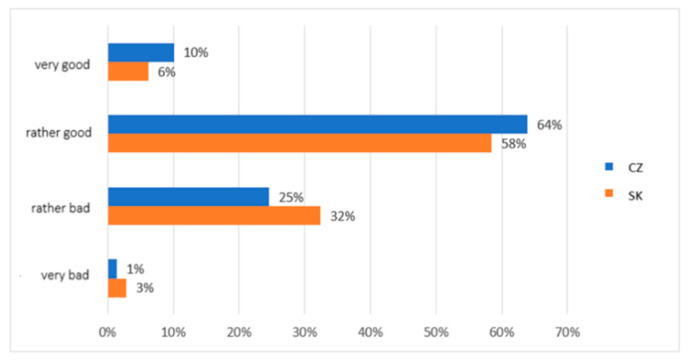
Descriptive statistics of the participants’ subjective perception of social and pastoral service quality (comparison of the healthy population of eastern Slovakia and north-western Czech Republic). Legend: CZ—healthy population of north-western Czech Republic, SK—healthy population of eastern Slovakia. Source: own resource.

We also asked about the subjective perception of the quality of service provided: How would you rate the quality of social and pastoral service in the society in which you live? It was possible to choose from answers on a scale from very good to very poor. The evaluation of the quality of social and pastoral service was very similar for both groups compared. A total of 6% of Slovaks and 10% of Czechs rated it as “very good” quality, and 58% of Slovaks and 64% of Czechs rated it as “rather good”. The quality of service at the “rather poor” level was assessed by 32% of Slovaks and 25% of Czechs and as “very poor” by 3% of Slovaks and 1% of Czechs (Figure 7).

**Figure 8:**
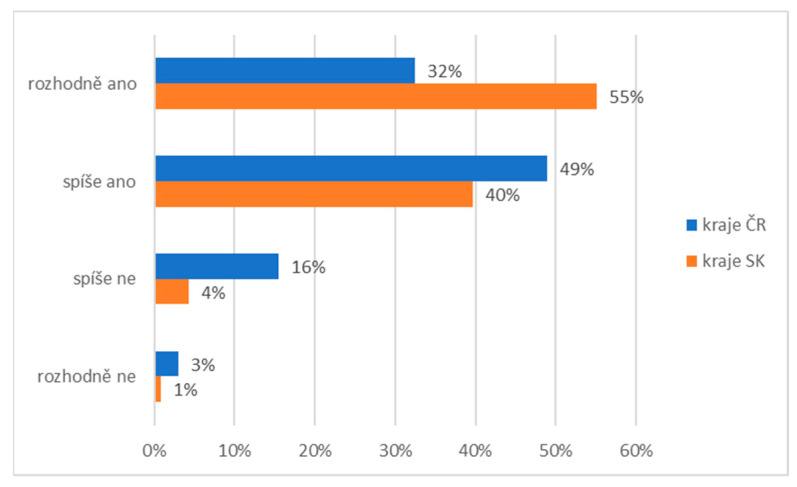
Descriptive statistics of the participants’ subjective perception of participation of the church in social and pastoral service (comparison of the healthy population of eastern Slovakia and north-western Czech Republic). Legend: CZ—healthy population of north-western Czech Republic, SK—healthy population of eastern Slovakia. Source: own resource.

In the penultimate question, we asked: Should the state, church institutions, or other associations become more involved in increased funding for this area? Research participants had to express their agreement with this statement on a 4-point scale. The results of the research in Figure 8 again show differences of opinion between the compared groups. The Slovaks perceived the church’s obligation to help people with a 55% absolute agreement rate and a 40% partial agreement rate. In the case of the Czechs, there was 32% absolute agreement and 49% partial agreement with this statement. Up to 16% of Czechs partially disagreed with this statement.

**Figure 9:**
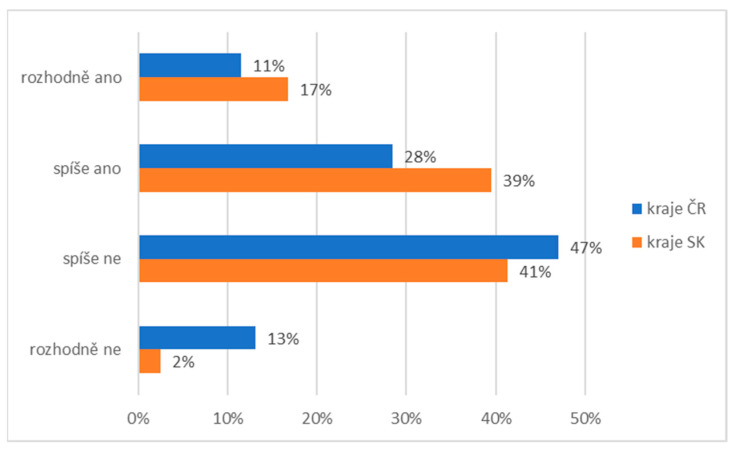
Descriptive statistics of the participants’ attendance in social and pastoral service (comparison of the healthy population of eastern Slovakia and north-western Czech Republic). Legend: CZ—healthy population of north-western Czech Republic, SK—healthy population of eastern Slovakia. Source: own resource.

In the last question, we were interested in the active participation of research participants in providing social and pastoral service. Our question was: Do you yourself participate in any way in the development and benefits of social service? Research participants had to express their agreement with this statement on a 4-point scale. There are also differences between research groups on this issue (Figure 9). A total of 17% of Slovaks and 11% of Czechs participated in social and pastoral services. In total, 39% of Slovaks and 28% of Czechs partially agreed with the statement, 41% of Slovaks and 47% of Czechs disagreed, and 2% of Slovaks and 13% of Czechs did not participate in these activities.

In order to obtain answers to our research question, which concerned differences in the research sample based on socio-demographic data, we subdivided them based on these variable participants in the verification of the differences in the individual features. As a result, this research section presents statistically significant differences calculated using Pearson’s chi-square test of the independence of variables in the contingency table.

### 3.1. Comparison of Selected Regions from the Czech Republic and Slovakia

The regions of the Czech Republic and Slovakia differ significantly statistically at the 1% level of significance in all the issues examined, except for the opinions on the quality of social services in the area. In both groups, respondents mostly answered that the quality is mostly rather good. Respondents from selected Slovak regions rated the need for social services with higher percentages than respondents from the Czech Republic (*p*-value of Pearson’s chi-square test: 0.000). In contrast to respondents from selected regions of the Czech Republic, respondents significantly more often stated that the social–pastoral service of religious organisations undoubtedly has an important social role (*p*-value of Pearson’s chi-square test: 0.000). They were also significantly more likely to report people in their area who volunteered for a church institution who were involved in the social assistance of others (*p*-value of Pearson’s chi-square test: 0.000) and were more likely to be involved in the development and benefit of social services (*p*—Pearson’s chi-square test value: 0.000).

### 3.2. Comparison by City or Village Size

There were statistically significant differences at the 5% level between groups of respondents divided according to the size of the city or village regarding the question about which form of assistance to socially vulnerable people is most effective (*p*-value of Pearson’s chi-square test: 0.013) and whether social–pastoral service religious organisations have a social role (*p*-value of Pearson’s chi-square test: 0.023). All groups stated that the most effective form of helping socially vulnerable people is family background. Respondents from villages and cities with more than 100,000 inhabitants (the group with the least and most inhabitants) stood out from the other groups. While other groups mentioned professional expertise in second place, respondents from cities with more than 100,000 inhabitants and villages cited spiritual assistance based on church institutions as a second option. Respondents from villages and towns with more than 100,000 inhabitants were also more likely than other groups to say that the socio-pastoral service of religious organisations undoubtedly played an important social role and that people in their neighbourhood were affiliated to some church institution that participated in social assistance to others.

### 3.3. Comparison by Gender

According to women, the need for social services in society is higher than for men (*p*-value of Pearson’s chi-square test: 0.025). Women were also more likely to report that the social–pastoral service of religious organisations undoubtedly plays a significant social role (*p*-value of Pearson’s chi-square test: 0.000). Conversely, men were more likely to report some form of social assistance to others (*p*-value of Pearson’s chi-square test: 0.002).

### 3.4. Comparison by Age

Regarding the age groups, respondents over 60 stated the highest percentages for expressing social services in society. On the contrary, respondents under 18 and between 18 and 25 stated (the *p*-value of Pearson’s chi-square test: 0.001) they had the lowest need for social services in society. According to respondents under 18, socially deviant individuals (people addicted to drugs, alcohol, psychotic aggression, etc.) deserve the most social attention (*p*-value of Pearson’s chi-square test: 0.000). However, people aged 18 and over often stated that the youngest and most vulnerable (children and adolescents in educational institutions) deserve the most social attention. Respondents under the age of 25 most often mentioned professional assistance and respondents from 26 and above mentioned family background (*p*-value of Pearson’s chi-square test: 0.000) as the most effective form of help for socially vulnerable people. Unlike other age groups, most respondents under the age of 18 did not find people in their area who participated in any church institution that participated in the social assistance of others (*p*-value of Pearson’s chi-square test: 0.000). Respondents under 18 were also no less involved in social assistance to others (*p*-value of Pearson’s chi-square test: 0.000).

## 4. Discussion

Our research aimed to obtain data on the differences between two groups of people who are territorially situated in the former state of Czechoslovakia. The study sought to point out the differences in the subjective perceptions of people in Central Europe who are mentally significantly close [24,25,26]. The study results related to the research were realised after the second wave of the pandemic situation of COVID-19. We intended to determine whether the study participants in eastern Slovakia and north-western Czech Republic differed in the type and level of subjective perception of need, quality, and participation in social and pastoral services. In addition, we were able to find differences in the perception of the church and church organisations and their role in society between different groups of the population. Therefore, the primary question of our research was to find out the differences in the level of subjective perceptions, needs, and participation in social and pastoral service between these two groups. A secondary issue in our research was to identify differences in the level of subjective perception, needs, and participation in social and pastoral service in the research sample based on gender, age, level of education, and the population of place of residence [27].

From the point of view of the importance and needs of social and pastoral services, it is clear from the research results that Slovaks from eastern Slovakia perceived this to be important much more subjectively than Czechs. For example, 31% of Slovaks subjectively perceived the need for social and pastoral service during a pandemic at a level of significance above 75%. However, compared to the Czechs, there was a significant difference of opinion of 12% in this case. This difference may be due to the historical events of the individual territories and the individual experiences of the individuals involved in the research. Prosocial behaviour is related to individual tendencies and the social and cultural environment [28] and is also related to individual tendencies and the context of the social and cultural environment. Therefore, cultural diversity can foster a positive attitude towards services to people [29,30,31]. A multicultural environment [32] also positively impacts on identity development, thus alleviating behavioural problems and protective effects against violence [33].

Identity development supports the building of the social skills of individuals. Sensitivity to the environment is part of a person’s ability to empathise and observe the consequences of socio-pathological phenomena in society [34,35]. Next in our study, the youngest children and adolescents were perceived by the participants as the most vulnerable groups during the pandemic. From the point of view of Slovaks, this group was marked in 40% of answers, and of Czechs in 39% of answers. For comparison, only 2% of research participants considered prisoners to be persons in need of social and pastoral service. This fact may point to the issue of criminal proceedings [36] and the social exclusion of this section of the population. Nevertheless, these people are perceived by the legislation as endangered and risky, and they need to receive attention in the process of providing social and pastoral services in order to be able to create a quality relationship in the social environment.

Building a firmly rooted relationship is a socio-psychological process necessary in creating a family. This process depends on social interactions that are present in every relationship. Through interactions, a person becomes a socially thinking and socially affected individual. Maybe this is why the participants in our research perceived family as key in several areas. Both groups in our research perceived the family environment as the reason for the emergence of socially deviant behaviour. Our research confirmed this fact by 48% of the answers of Slovak respondents and 49% of the answers of Czech respondents. In this part of our research, we identified the first fundamental difference in the subjectively perceived absence of religious influence between the groups. Relationship models and attachment patterns begin to take shape in early childhood [37]. Many mental disorders also have their roots during this period. In addition to social deviations, many mental illnesses have their origins in early childhood and are influenced by family.

An example is schizophrenia, a multisystem impairment of brain function that is often conditioned by social and genetic factors and associated with a disorganised attachment [38]. Anxiety disorders are often associated with anxiety. Dysfunctions of the emotional regulatory system in affective disorders, such as depression [39] or mania, result from injured relationship interactions during the underlying depressive conflict [40,41]. Research into the adult population [42] points to a connection between perfectionism, burnout, irrational beliefs [43], and the neurotic form of altruism, which is one of the forms of pathological management of fundamental depressive conflict.

In our study, up to 20% of Slovaks and only 5% of Czechs indicated the importance of religion, faith, and the church in preventing deviant behaviour. Therefore, we can contemplate what role these areas of human life can play in living an emotionally corrective experience. The fact is that religious pluralism in European countries has not achieved the expected weakening of faith. On the contrary, it has been shown to lead to an overall increase in religiosity. The surprising return of interest in religion, on the other hand, manifested itself in its fate—with the globalisation of the world, the globalisation of religion and the activity of religious radicalism and fundamentalism increased, which led to a reaction in the structures of society but also in everyday life and interpersonal relationships [44]. The results of the study show that Slovaks perceive spirituality and the church to be more significant than Czechs. The social situation is significantly influenced by the media, and our research participants also perceived this fact [12,44].

The differences between the research groups in this area were repeated in another of our research questions. Again, family background was a dominant factor in possibilities and support for vulnerable individuals and groups. In the group of Slovaks, 45% of people had this opinion, and in the group of Czechs, 39% of the respondents stated this. However, the importance of spiritual assistance based on church institutes was identified only by research participants from Slovakia in the range of 29% of responses (only 6% in Czechs). Again, cultural and religious differences are evident in the two groups. Additionally, regarding the social role of religious organisations, as many as 93% of Slovaks in our study see the church’s role in the social and pastoral service. The church and church organisations played a significant social role during the pandemic. Many people in the social and pastoral service profess membership of the church. However, as many as 63% of Slovaks and only 26% of Czechs noticed specific activities of the church. A total of 35% of research participants from Czech Republic did not notice any church activity during the pandemic in the field of social and pastoral service. At the same time, however, the quality of the services provided was perceived by both groups almost equally.

Helping is an integral part of culture [45] and religion in Central Europe [46]. Experts and the general public are interested in why people behave either pro-socially or not in certain circumstances. Many people place responsibility for pro-social behaviour on others and avoid it themselves. The diffusion of responsibility [47] can be critical in a pandemic situation saturated with social isolation [48]. This isolation is caused by restrictive measures in individual states, limiting the population’s community activities. More than 55% of Slovaks and 32% of Czechs in our research had a strong subjective perception of the vital need for state and church participation in social and pastoral services. Pro-social behaviour is a complex phenomenon, and it is necessary to realise that part of it is also related to an individual’s motives. These are relatively permanent preconditions for helping in a situation that requires it. These characteristics are usually acquired during childhood. Therefore, it is not appropriate to think that responsibility for providing social and pastoral service is an institutional matter. Each institution is made up of individual people. The individual feels a commitment and an obligation to act in a critical situation depending on whether the act itself supports or, conversely, disproves its fundamental values [49,50].

The problem is connected with the lack of interest in transcendent values, with partial identification with some truths of faith and moral values. Sometimes, we see so-called parallel identification in this colourful but erroneous, self-mixed cocktail of our beliefs. Here, unfortunately, we sometimes see practicing Christians who believe in the resurrection of Christ, but at the same time enthusiastically complete initiations in reiki, or card divination, which, however, have the impression that Christians are like them. They lack (and we also have) the courage to believe in following Christ as D. Bonhoeffer or S. Kierkegaard did [51,52,53,54].

Today has brought various other pitfalls that may be challenges. We have freedom here, in that no one persecutes us anymore for expressing our religious opinion or faith. We can build churches and establish nursing homes—hospices, where clergy can serve the dying or those who need spiritual and social help. However, on the other hand, we have an economically healthy environment, advanced technologies, along with a high degree of globalisation that seeks to satisfy material hunger and desire. One thus stands at “Kierkegaard’s” crossroads “either–or” to be and to live or to have and to own. This period has shown us all how weak, dependent, and at the same time, without (power) man is [55,56].

As part of a particular social group and community, people find help from others daily. The type of help can take various forms, whether a circulating good deed or a risk to one’s own life for another person’s good. There is human potential for the right to manage in such cases, a willingness to help [57]. Therefore, pro-social behaviour is an important dimension of social competence [58,59]. The value of helping is part of personal well-being [60,61]. At the same time, a pro-social person feels inner satisfaction from the act performed. This principle can also be demonstrated in the results of our research. In our research, more than 56% of Slovaks and 45% of Czechs actively participated in social and pastoral services during the pandemic. Pro-social behaviour manifests itself in everyday life situations or the dramatic, emotionally demanding moments experienced during the COVID-19 pandemic.

## 5. Conclusions

Based on the results of our research, we can conclude that eastern Slovaks subjectively perceive a higher need for social and pastoral service in the pandemic than north-western Czechs. At the same time, eastern Slovaks and north-western Czechs do not differ in the perception of social and pastoral service, and almost all groups refer equally in terms of the need for “social attention”. Furthermore, we can see that almost half of all participants think that the cause of deviant behaviour is an unsuitable family environment and also that family is a key supportive factor in preventing deviant behaviour. The key difference appears to be the perception of the church and religious organisations in the current activities of social and pastoral services carried out during the COVID-19 pandemic situation. There are also differences between these groups in the subjective perception of involving the church and the state in serving the population. The limitation of this study is based on the culturally conditioned environment that can be affected by the affiliation of people to the church. There is also a question about the participants’ motivation in the research, which can distort our results. Therefore, it would be appropriate to verify the data obtained by further research using standardised questionnaires, bringing different results in cultural and psychological disciplines. In conclusion, we would like to recommend our results for further investigation of cultural, social, individual, psychological, and religious differences in parts of Slovakia and the Czech Republic or other Central European countries and possibly in the context of the broader European and non-European context.

## Data Availability

Not applicable.
